# Differential In Vitro Inhibition of Thrombin Generation by Anticoagulant Drugs in Plasma from Patients with Cirrhosis

**DOI:** 10.1371/journal.pone.0088390

**Published:** 2014-02-04

**Authors:** Wilma Potze, Freeha Arshad, Jelle Adelmeijer, Hans Blokzijl, Arie P. van den Berg, Joost C. M. Meijers, Robert J. Porte, Ton Lisman

**Affiliations:** 1 Surgical Research Laboratory, University of Groningen, University Medical Center Groningen, Groningen, The Netherlands; 2 Department of Gastroenterology, University of Groningen, University Medical Center Groningen, Groningen, The Netherlands; 3 Department of Experimental Vascular Medicine, Academic Medical Center, University of Amsterdam, Amsterdam, The Netherlands; 4 Section of Hepatobiliary Surgery and Liver Transplantation, Department of Surgery, University of Groningen, University Medical Center Groningen, Groningen, The Netherlands; Institut National de la Santé et de la Recherche Médicale, France

## Abstract

**Background:**

Treatment and prevention of thrombotic complications is frequently required in patients with cirrhosis. However anticoagulant therapy is often withheld from these patients, because of the perceived bleeding diathesis. As a result of the limited clinical experience, the anticoagulant of choice for the various indications is still not known.

**Objectives:**

We evaluated the in vitro effect of clinically approved anticoagulant drugs in plasma from patients with cirrhosis.

**Patients/Methods:**

Thirty patients with cirrhosis and thirty healthy controls were studied. Thrombin generation assays were performed before and after addition of unfractionated heparin, low molecular weight heparin, fondaparinux, dabigatran, and rivaroxaban, to estimate anticoagulant potencies of these drugs.

**Results:**

Addition of dabigatran led to a much more pronounced reduction in endogenous thrombin potential in patients compared to controls (72.6% reduction in patients vs. 12.8% reduction in controls, P<0.0001). The enhanced effect of dabigatran was proportional to the severity of disease. In contrast, only a slightly increased anticoagulant response to heparin and low molecular weight heparin and even a reduced response to fondaparinux and rivaroxaban was observed in plasma from cirrhotic patients as compared to control plasma.

**Conclusions:**

The anticoagulant potency of clinically approved drugs differs substantially between patients with cirrhosis and healthy individuals. Whereas dabigatran and, to a lesser extent, heparin and low molecular weight heparin are more potent in plasma from patients with cirrhosis, fondaparinux and rivaroxaban showed a decreased anticoagulant effect. These results may imply that in addition to dose adjustments based on altered pharmacokinetics, drug-specific dose adjustments based on altered anticoagulant potency may be required in patients with cirrhosis.

## Introduction

Chronic liver disease has long been considered as the epitome of acquired bleeding disorders, due to clinically observed bleeding complications (e.g. variceal bleeding) in combination with a decreased number and function of platelets, decreased synthesis of coagulation factors by the diseased liver, and hyperfibrinolysis[1]. Conventional coagulation tests such as the prothrombin time (PT) and activated partial thromboplastin time (APTT), designed to assess isolated defects of pro-coagulants, but insensitive for anticoagulant factors, are frequently prolonged in patients with cirrhosis suggesting defective hemostasis and thus a bleeding tendency. Recently, more sophisticated tests of hemostasis that allow assessment of the true balance between pro- and anticoagulant factors, have been used to reassess the hemostatic capacity of patients with liver disease. In particular, thrombin generation testing performed in the presence of thrombomodulin (TM) has demonstrated normal or even superior thrombin generation compared to healthy volunteers [2]–[7]. These experiments in conjunction with clinical observations have led to the concept of ‘rebalanced hemostasis’, which suggests hemostatic balance by a concomitant decrease in both pro- and anticoagulant drivers [8], [9]. Although the hemostatic system of patients with liver disease is in a ‘rebalanced’ status, clinical thrombotic events and bleeding complications suggest that this balance is more unstable as compared to the balance in healthy individuals and can be easily tipped over to a hyper- or a hypocoagulable state [10].

Despite historical beliefs that patients with liver disease are ‘auto-anticoagulated’, thrombotic complications do occur in cirrhotic patients and form evidence for hypercoagulability in these patients [11], [12]. Because of the perceived bleeding diathesis of liver disease, prophylactic anticoagulant therapy is presumably underused in patients with cirrhosis. Furthermore, treatment of thrombotic complications is frequently required, as patients with liver disease can suffer from deep vein thrombosis or pulmonary embolism, and portal vein thrombosis [11], [12]. Moreover, patients may require anticoagulation for systemic arterial events [12].

Nowadays, there is increasing recognition of various thrombotic complications that may occur in patients with chronic liver disease and therefore an increase in the use of anticoagulant therapy in these patients may be expected. Due to the limited clinical experience, the anticoagulant of choice for the various indications is still unclear. Vitamin K antagonists have major drawbacks when used in cirrhotic patients, as vitamin K antagonist therapy requires monitoring by the international normalized ratio (INR) which is frequently already abnormal in cirrhotic patients. Clinical data on the use of low molecular weight heparin (LMWH) indicate that the drug is safe and effective in both the treatment and prevention of portal vein thrombosis [13], [14]. In addition heparins appear safe and effective in prevention of venous thrombosis [15]. However, the mode of administration of these agents as well as the concern for heparin-induced thrombocytopenia (HIT) may limit long-term use. In addition, monitoring of heparins is complicated by the substantial underestimation of heparin levels when tested by an anti-Xa assay [16]–[18]. Finally, LMWH accumulation is known to occur in patients with renal failure, and thus patients with chronic liver disease and decreased renal function likely require dose-adjustments based on altered pharmacokinetics.

There is little published clinical experience with the new oral anticoagulants (NOACs) such as the direct factor Xa and thrombin inhibitors Rivaroxaban and Dabigatran in patients with a chronic liver disease. However, these new agents would be potentially applicable in both long- and short term anticoagulant strategies in patients with cirrhosis, and such agents have (theoretical) advantages over currently used strategies [19]. Nevertheless, since currently available NOACs such as Rivaroxaban and Dabigatran are cleared by liver and kidneys, it is conceivable that the pharmacokinetics of these drugs is also altered in patients with (advanced) liver disease.

In vitro studies have demonstrated that LMWH has a more profound anticoagulant effect in plasma from patients with cirrhosis as compared to plasma from healthy controls [17]. Possible increased responses to anticoagulant drugs may have important consequences for the dosing regiments in these patients. In this study, we aimed to investigate the efficacy of currently approved and widely used anticoagulant drugs by performing *in vitro* thrombin generation tests in plasma of patients with cirrhosis.

## Patients and Methods

### Patients

Thirty adult patients with a previous clinical diagnosis of cirrhosis, who were under routine control for their disease by the department of Hepatology of the UMCG or who were admitted at the Hepatology ward of the UMCG, were included in the study. The patients were classified according to the Child-Pugh classification [20]. Ten patients with Child's A cirrhosis, 10 patients with Child's B cirrhosis, and 10 patients with Child's C cirrhosis were studied. Exclusion criteria were documented history of congenital coagulation disorders, presence of active infection (<2 weeks), presence of acute liver failure, use of anticoagulant drugs in the past 10 days, pregnancy, HIV positivity, and recent (<7 days) transfusion with blood products.

The control group consisted of thirty adult healthy volunteers working at our institution. Exclusion criteria for the control group were documented history of congenital coagulation disorders, documented history of hepatic disease, recent viral infection (<2 weeks), use of anticoagulant drugs in the past 10 days, pregnancy, and HIV positivity.

### Ethics statement

This study protocol was approved by the medical ethical committee of the University Medical Center Groningen, Groningen, The Netherlands and written informed consent was obtained from each subject before inclusion in the study. The study was conducted according to the principles expressed in the Declaration of Helsinki.

### Plasma samples

Blood samples from each patient and control were drawn by clean vena-puncture and collected into vacuum tubes containing 3.8% trisodium citrate as an anticoagulant, at a blood to anticoagulant ratio of 9∶1. Platelet poor plasma was prepared by double centrifugation at 2000 g and 10.000 g respectively for 10 min. Plasma was snap-frozen and stored at −80°C until use.

### Addition of anticoagulants to plasma samples

The following anticoagulants were added to plasma samples of every cirrhotic patient and control. The mentioned concentrations represent final concentrations in plasma.

Unfractionated heparin (Leo Pharma, Denmark), 0.1 U/ml
The LMWH Clexane (Sanofi-Aventis BV, Gouda, the Netherlands), 0.2 U/ml
Fondaparinux (Arixtra) (GlaxoSmithKline BV, Zeist, the Netherlands), 0.5 µg/ml
Dabigatran (Alsachim, Illkirch Graffenstaden, France), 300 ng/ml
Rivaroxaban (Alsachim, Illkirch Graffenstaden France), 25 ng/ml

The final concentrations of the various drugs were based on initial experiments in which drugs were added in various concentrations to pooled normal plasma after which thrombin generation was performed as described in the next paragraph. Those drugs concentrations which gave appreciable (but not maximal) inhibition of thrombin generation in pooled normal plasma were selected so it would be possible to detect both increased and decreased drug effects in patients compared to controls.

### Thrombin generation

The thrombin generation test was performed using platelet-poor plasma (PPP) with the fluorimetric method described by Hemker, Calibrated Automated Thrombography® (CAT) [21]. Coagulation was activated using commercially available reagents containing recombinant tissue factor (TF, final concentration 5 pM), phospholipids (final concentration 4 µM), in the presence or absence of soluble thrombomodulin (TM, the concentration of which is not revealed by the manufacturer). These reagents were purchased from Thrombinoscope BV, Maastricht, The Netherlands. Thrombin Calibrator (Thrombinoscope BV) was added to calibrate the thrombin generation curves. A fluorogenic substrate with CaCl_2_ (FluCa-kit, Thrombinoscope BV, Maastricht, The Netherlands) was dispensed in each well to allow a continuous registration of thrombin generation. Fluorescence was read in time by a fluorometer, Fluoroskan Ascent® (ThermoFisher Scientific, Helsinki, Finland). All procedures were according to the protocol suggested by Thrombinoscope B.V.

The anticoagulant potency of the different drugs was expressed as the percentual reduction of endogenous thrombin potential (ETP), peak, or velocity index, and the percentual increase in lag time after the addition of anticoagulants. These percentages were compared between patients and controls.

### Routine coagulation laboratory tests

The INR was assessed with commercially available methods on an automated coagulation analyzer (ACL 500 TOP) with reagents (Recombiplastin 2G) and protocols from the manufacturer (Instrumentation Laboratory, Breda, the Netherlands). Levels of factor (F) VIII, II, and X, and antithrombin (AT) were measured on an automated coagulation analyzer (ACL 500 TOP) with reagents and protocols from the manufacturer (Recombiplastin 2G for FII and FX, Hemosil (R) SynthASil for FVIII, and Liquid Antithrombin reagent for AT) (Instrumentation Laboratory). Total protein S antigen was assayed by enzyme-linked immunosorbent assay (ELISA) using antibodies from DAKO (Glostrup, Denmark). Free protein S was measured by precipitating the C4b-binding protein-bound fraction with polyethylene glycol 8000 and measuring the concentration of free protein S in the supernatant. Protein C was determined using the Coamatic protein C activity kit from Chromogenix (Mölndal, Sweden)

### Statistical analysis

Data are expressed as means (with standard deviations (SDs)), medians (with interquartile ranges), or numbers (with percentages) as appropriate. Means of two groups were compared by Student's t-test or Mann-Whitney U test as appropriate. Multiple groups were compared using One-way ANOVA (with the Bonferroni posttest) or Kruskal-Wallis H test (with Dunn's posttest) as appropriate. Spearman's correlation coefficient was used to assess correlation between continuous variables. P values of 0.05 or less were considered statistically significant. GraphPad Prism (San Diego, USA) and IBM SPSS Statistics 20 (New York, USA)) were used for analyses.

## Results

### Patient characteristics

The main characteristics of the study population are reported in Table 1. Thirty patients with cirrhosis (18 males and 12 females) were included, and they were categorized according to the severity of liver disease as expressed by the Child Pugh classes (10 Child A, 10 Child B and 10 Child C patients). Thirty healthy subjects (14 males and 16 females) were included as controls. The most common etiology of liver disease was alcoholic, especially in the Child class C patients.

**Table 1:** Demographic and clinical characteristics of the study population.

	Cirrhotic patients			P-value
	Child A	Child B	Child C	
	n = 10	n = 10	n = 10	
**Characteristics**				
**MELD score**	8.0 [6.0–10.0]	11.5 [8.0–19.0]	15.5 [6.0–19.0]	.002
**Age (yrs)**	56.0 [14.2]	50.5 [12.5]	54.9 [8.3]	.560
**Sex (male)**	4 (40)	6 (60)	8 (80)	.248
**BMI**	26.0 [18.8–31.4]	26.5 [22.0–41.4]	26.3 [22.2–36.3]	.705
**Smoking (number)**	4 (40)	1 (10)	4 (40)	.297
**Alcohol (U per week)**	0 [0–1]	0 [0–7]	0 [0–490]	.670
**Etiology of liver disease**				
** Alcoholic**	2 (20)	1 (10)	10 (100)	<.001
** HCV**	1 (10)	0 (0)	0 (0)	1.000
** NASH**	1 (10)	2 (20)	0 (0)	.754
** Hemochromatosis**	0 (0)	1 (10)	0 (0)	1.000
** PBC**	1 (10)	0 (0)	0 (0)	1.000
** PSC**	2 (20)	2 (20)	0 (0)	.507
** Auto-immune**	3 (30)	1 (10)	0 (0)	.286
** Alcoholic + NASH**	0 (0)	1 (10)	0 (0)	1.000
** Unknown**	0 (0)	2 (20)	0 (0)	.310
**Co-morbidity**				
** Cardiovascular**	3 (30)	2 (20)	0 (0)	.321
** DM**	2 (20)	1 (10)	1 (10)	1.000
**Plasma levels**				
** Serum bilirubin (μmol/L)**	15 [5–35]	40 [18–61]	82 [46–131]	<.0001
** Serum albumin (g/L)**	36 [28–44]	33 [27–63]	27 [25–91]	.003
** Serum creatinin (μmol/L)**	69 [23]	72 [32]	70 [20]	.966
** Hemoglobin (mmol/L)**	8.2 [1.4]	7.2 [0.9]	6.5 [0.8]	.005
** Leukocytes (10^9^/L)**	6.5 [4.5]	5.1 [2.1]	7.4 [5.3]	.463
** Platelets (10^9^/L)**	112 [16–258]	86 [28–471]	76 [44–165]	.565

HCV: Hepatitis C virus, NASH: Non-alcoholic steatohepatitis, PBC: Primary biliary cirrhosis, PSC: Primary sclerosing cholangitis, DM: Diabetes Mellitus.

Data are expressed as number (%), mean [SD], or median [range].

INR, FVIII, FII, FX, AT, protein S and protein C levels are shown in Table 2. Patients with cirrhosis showed a statistically significant prolongation of INR and a decrease in all measured coagulation proteins, except for FVIII (which was increased), as compared to controls. The reduction in levels of plasmatic factors was proportional to the severity of liver disease.

**Table 2:** Coagulation parameters in cirrhotic patients and controls.

	Cirrhotic patients			Healthy controls	P-value
	Child A	Child B	Child C		
**INR**	1.1 [0.9–1.2]	1.2 [1.0–2.0]	1.5 [1.4–1.7]	1.0 [0.9–1.1]	<.0001
**FVIII (%)**	132 [92–185]	129 [91–254]	140 [108–199]	94 [56–138]	<.0001
**FII (%)**	83 [50–104]	69 [43–98]	42 [27–71]	107 [88–127]	<.0001
**FX (%)**	90 [74–136]	81 [41–193]	56 [45–69]	106 [80–147]	<.0001
**AT (%)**	80 [42–107]	50 [27–113]	40 [23–45]	106 [87–126]	<.0001
**Protein C (%)**	71 [35–105]	53 [22–155]	27 [12–44]	109 [87–168]	<.0001
**Protein S total (%)**	76 [61–109]	66 [35–200]	57 [52–78]	90 [68–121]	.002
**Protein S free (%)**	75 [59–130]	69 [45–105]	82 [56–110]	99 [71–136]	.002

INR: International normalized ratio, AT: Antithrombin.

Data are expressed as median [range].

### Thrombin generation

When thrombin generation was performed without addition of any anticoagulant, the endogenous thrombin potential (ETP) and peak thrombin generation in plasma from cirrhotic patients were comparable to that of healthy controls, both in absence and presence of TM. The ETP is the presence of TM was slightly, but significantly higher in patients compared to controls. Values in the absence of TM were: ETP 872±260 nM*min (mean +/− SD), peak 169±41 nM in patients vs. ETP 945±268 nM*min, peak 188±49 nM in healthy controls, P = 0.28 for ETP, P = 0.11 for peak; in the presence of TM: ETP 677±304 nM*min, peak 146±48 nM in patients vs. ETP 510±259 nM*min, peak 131±53 nM in controls, P = 0.03 for ETP, P = 0.26 for peak.

**Figure 1:**
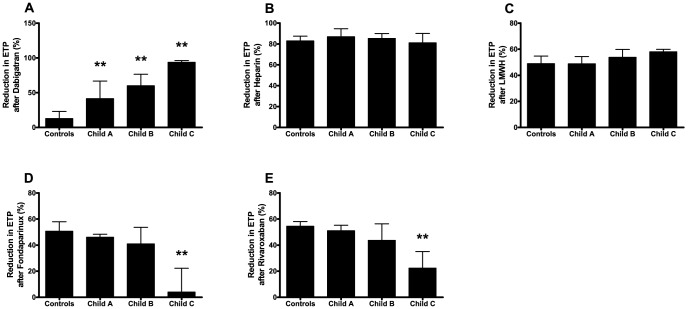
Reductions in ETP after the addition of the different anticoagulant drugs. Reduction in ETP after the addition of (A) dabigatran, (B) heparin, (C) LMWH, (D) Fondaparinux, and (E) rivaroxaban in plasma from patients with Child A, B, and C cirrhosis and healthy controls. Bars indicate medians with the error bars representing interquartile ranges. ** = P<0.01 compared to controls.

To study the anticoagulant potency of antithrombotic drugs in plasma from cirrhotic patients, percentual reductions in ETP, peak, velocity index, and percentual increases in lag time after the addition of the different anticoagulants were calculated and compared between plasma from cirrhotic patients and controls. All data are shown in table 3. In figure 1, the reduction in ETP performed in the presence of TM after the addition of the different anticoagulants in plasma from patients and controls is shown. A detailed analysis of the results for each drug is given below.

**Table 3:** Inhibition of in vitro thrombin generation after addition of various anticoagulant drugs to plasma taken from patients with cirrhosis or plasma from healthy controls.

	**Dabigatran**							
	TM-				TM+			
	ETP	Peak	Lag-time	Velindex	ETP	Peak	Lag-time	Velindex
Controls	45.4 [39.4–47.7]	29.8 [22.0–34.9]	207.8 [143.0–267.1]	3.8 [−11.1–17.3]	12.8 [−10.5–23.0]	0.2 [−24.3–18.9]	199.7 [141.5–258.5]	−15.7 [−46.2–8.9]
All patients	75.2 [62.5–92.2]**	83.1 [64.5–96.6]**	165.4 [133.2–299.4]	84.9 [63.3–98.3]**	72.6 [39.1–90.9]**	82.9 [56.8–96.3]**	165.4 [136.4–301.8]	90.3 [62.1–98.2]**
Child A	64.3 [54.4–70.1]**	67.6 [46.8–79.9]**	245.3 [164.3–393.0]	66.7 [29.4–82.3]**	41.4 [32.4–66.8]**	55.3 [31.5–79.6]**	276.0 [154.3–402.8]	66.1 [26.8–83.0]**
Child B	67.1 [60.5–79.3]**	69.8 [63.4–92.5]**	160.1 [125.6–436.2]	72.7 [57.5–95.0]**	59.9 [28.3–76.4]**	72.7 [57.4–92.3]**	155.7 [136.0–493.9]	89.8 [57.6–95.2]**
Child C	93.7 [81.2–96.4]**	97.2 [92.9–97.8]**	152.1 [84.8–185.0]	98.3 [95.3–98.5]**	93.7 [78.1–96.0]**	97.1 [92.1–97.9]**	155.0 [75.2–175.9]	98.5 [94.8–98.7]**
	**Heparin**							
	TM-				TM+			
	ETP	Peak	Lag-time	Velindex	ETP	Peak	Lag-time	Velindex
Controls	46.0 [32.8–57.2]	81.3 [64.0–86.9]	50.0 [33.5–67.6]	91.3 [84.9–95.9]	83.0 [75.1–87.5]	87.3 [78.9–91.3]	39.2 [19.8–47.3]	88.3 [79.8–92.5]
All patients	62.0 [54.1–76.8]**	82.3 71.3–92.6]	58.4 [29.8–78.0]	92.8 [85.5–96.3]	84.6 [79.4–91.0]	87.4 [78.6–93.0]	39.9 [16.9–69.1]	90.6 [84.0–95.5]
Child A	59.8 [46.2–81.1]	82.5 [76.3–94.3]	43.7 [29.8–89.9]	93.5 [90.4–97.4]	86.9 [79.9–94.7]	89.9 [83.0–96.5]	30.0 [19.2–68.0]	92.9 [85.7–97.4]
Child B	62.0 [46.7–82.0]	81.9 [63.5–93.8]	67.2 [21.6–78.3]	92.6 [82.4–98.1]	85.2 [81.1–90.0]	87.4 [81.8–91.8]	45.0 [7.2–70.0]	90.6 [84.8–92.8]
Child C	65.5 [57.2–71.2]*	76.1 [71.3–89.8]	59.1 [32.0–82.4]	87.1 [84.9–95.8]	81.1 [77.5–90.1]	81.1 [75.8–92.7]	45.0 [19.0–72.8]	86.9 [79.7–95.5]
	**LMWH**							
	TM-				TM+			
	ETP	Peak	Lag-time	Velindex	ETP	Peak	Lag-time	Velindex
Controls	32.8 [29.8–39.0]	43.3 [33.1–52.1]	10.5 [6.9–17.7]	52.9 [42.1–61.3]	48.9 [44.3–54.8]	47.5 [41.1–53.5]	12.1 [0.0–19.8]	49.6 [38.1–56.2]
All patients	42.1 [37.5–51.5]**	37.0 [32.4–43.5]	7.7 [0.0–21.2]	37.1 [33.0–47.5]**	52.3 [46.3–60.0]	41.6 [34.8–46.6]	10.4 [0.0–20.8]	40.0 [31.9–48.2]*
Child A	38.8 [31.8–42.2]	37.1 [30.6–45.0]	6.6 [0.0–21.2]	43.0 [30.8–50.0]	48.8 [44.3–54.3]	43.5 [36.5–49.9]	10.9 [4.4–19.8]	43.4 [36.5–52.0]
Child B	41.3 [37.1–49.1]]*	37.9 [31.9–49.0]	7.7 [0.0–25.6]	39.9 [34.5–51.3]	53.7 [42.9–59.8]	42.0 [34.3–50.1]	0.0 [0.0–25.6]	42.5 [26.5–51.7]
Child C	51.9 [42.2–55.9]**	36.2 [32.8–41.0]	12.8 [0.0–19.4]	34.1 [30.3–37.5]**	58.0 [48.6–59.9]	39.9 [34.5–45.8]	17.3 [0.0–25.6]	38.4 [27.0–43.1]
	**Fondaparinux**							
	TM-				TM+			
	ETP	Peak	Lag-time	Velindex	ETP	Peak	Lag-time	Velindex
Controls	5.2 [0.8–20.2]	37.3 [17.3–49.2]	28.2 [21.4–35.6]	60.5 [43.0–67.3]	50.6 [42.4–57.9]	54.4 [47.7–62.3]	19.8 [12.3–25.6]	62.2 [52.8–67.7]
All patients	−2.0 [−9.3–5.5]**	14.5 [7.8–30.8]**	26.9 [25.2–39.5]	38.3 [24.4–55.8]**	32.0 [5.6–47.4]**	36.8 [14.6–52.3]**	25.6 [19.8–28.9]**	42.8 [21.8–60.1]**
Child A	−0.2 [−4.0–6.2]	27.5 [16.2–37.7]	39.2 [30.9–40.5]	54.6 [39.0–57.1]	46.1 [37.8–48.4]	49.0 [44.1–55.3]	23.5 [19.8–29.0]	55.5 [52.3–61.5]
Child B	−2.0 [−6.5–14.6]	18.1 [8.3–40.2]	25.6 [18.9–37.4]	45.1 [26.2–61.2]	41.0 [13.8–53.7]	44.2 [19.4–58.7]	25.6 [15.8–25.6]	53.1 [26.1–64.2]
Child C	−8.0 [−11.2–1.4]**	8.5 [5.2–12.2]**	25.6 [25.5–35.7]	26.7 [17.0–31.0]**	3.9 [1.4–22.2]**	12.9 [8.6–22.5]**	25.6 [25.0–38.1]**	20.9 [13.0–33.2]**
	**Rivaroxaban**							
	TM-				TM+			
	ETP	Peak	Lag-time	Velindex	ETP	Peak	Lag-time	Velindex
Controls	3.2 [−1.5–7.9]	47.6 [39.0–55.6]	59.5 [49.6–64.8]	75.1 [67.6–80.4]	54.5 [50.4–58.1]	61.8 [55.7–64.3]	39.5 [26.2–48.2]	68.4 [64.0–71.5]
All patients	2.5 [−4.6–6.2]	30.7 [15.6–42.9]**	64.2 [50.1–75.2]	55.2 [38.9–69.1]**	39.3 [19.7–53.2]**	46.5 [27.1–60.5]**	48.2 [36.7–56.7]*	57.3 [35.7–70.4]**
Child A	1.0 [−4.1–5.8]	39.0 [26.9–47.7]	60.8 [56.2–75.5]	66.3 [51.3–77.5]	51.1 [43.4–55.2]	60.2 [47.7–63.1]	47.5 [39.5–50.3]	68.5 [56.6–72.6]
Child B	4.3 [−5.3–9.5]	32.9 [15.3–47.1]	66.9 [57.3–76.0]	61.6 [38.2–72.1]*	43.7 [18.6–56.3]	51.0 [26.2–63.2]	50.4 [30.8–56.7]	59.6 [35.1–71.7]
Child C	−0.2 [−7.3–6.5]	17.1 [15.0–23.3]**	50.6 [42.3–81.2]	38.5 [31.4–51.2]**	22.3 [11.6–35.1]**	28.3 [21.1–39.5]**	49.3 [36.7–77.0]	38.4 [30.0–49.8]**

TM: thrombomodulin, ETP: Endogenous thrombin potential, Velindex: velocity index, LMWH: low molecular weight heparin.

Shown are the percentual inhibition of the ETP, peak, or velocity index, and the percentual increase in the lag time. Data are expressed as median percentages with interquartile range.

* = P<0.05, ** = P<0.01 compared to controls.

### Dabigatran

When dabigatran was added to plasma of healthy controls, the ETP was reduced by 45.4 [39.4–47.7]% (median [IQR]) and peak thrombin generation by 29.8 [22.0–34.9]%. In contrast, the ETP and peak hardly decreased in the presence of TM (12.8 [−10.5–23.0]% reduction in ETP and 0.2 [−24.3–18.3]% reduction in peak). The lag time was substantially prolonged after addition of dabigatran both in absence and presence of TM (Table 3).

Addition of dabigatran led to a much more pronounced reduction in peak thrombin generation in patients compared to controls, both in absence (83.1 [64.5–96.6]% reduction in patients vs. 29.8 [22.0–34.9]% reduction in controls, P<0.0001) and the presence of TM (82.9 [56.8–96.3]% reduction in patients vs. 0.2 [−24.3–18.3]% reduction in controls, P<0.000001). The decrease in thrombin generation with dabigatran mirrored the severity of liver disease. Child class C patients exhibited the most pronounced reduction in peak thrombin generation, with a median reduction of 97.1 [92.1–97.9]% when tested in the presence of TM.

**Figure 2:**
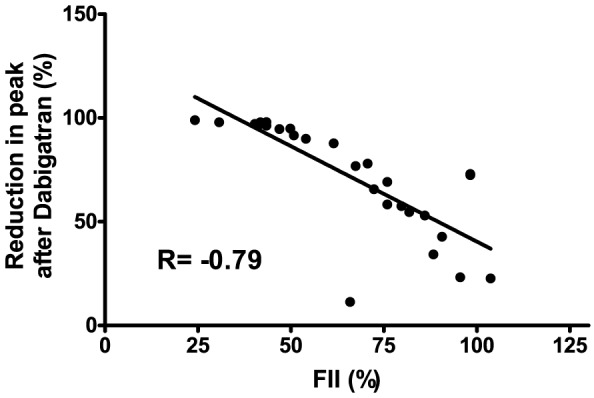
Correlation between FII levels and reductions in thrombin generation after dabigatran. Correlation between plasmatic FII levels and percentual reduction of peak thrombin values when tested in the presence of TM after addition of 300/ml dabigatran in plasma from patients with cirrhosis.

Interestingly, reductions in thrombin generation by dabigatran when tested in the presence of TM strongly correlated with plasma prothrombin (FII) levels (r = −0.80, P<0.0001) (Figure 2).

### Heparin

After the addition of unfractionated heparin the reduction of thrombin generation was more profound in plasma from cirrhotic patients compared to controls, with a median ETP reduction of 62.0 [54.1–76.8]% in patients vs. 46.0 [32.8–57.2]% in controls (P = 0.0003) (Table 3). However, in the presence of TM the reductions in ETP were not significantly different in plasma from cirrhotic patients compared to controls. The decrease in peak thrombin generation was comparable between cirrhotic patients and controls (87.4 [78.6–93.0]% in patients vs. 87.3 [78.9–91.3]% in controls, P = 0.46, when tested in the presence of TM).

### Low molecular weight heparin

After the addition of LMWH the decrease in the ETP in the absence of TM was more pronounced in plasma from cirrhotic patients compared to controls (42.1 [37.5–51.5]% reduction in patients vs. 32.8 [29.8–39.0]% reduction in controls, P<0.001) (Table 3). The reduction in peak thrombin generation by LMWH was not significantly different in plasma from cirrhotic patients (37.0 [32.4–43.5]% reduction) compared to controls (43.3 [33.1–52.1]% reduction) (P = 0.14). Moreover, when thrombin generation was performed in the presence of TM there was no difference in ETP reduction in plasma from patients compared to controls (52.3 [46.3–60.0]% reduction in patients vs. 48.9 [44.3–54.8]% reduction in controls, P = 0.05).

### Fondaparinux

Addition of fondaparinux in the absence of TM hardly affected the ETP in both patients and controls. The reduction in thrombin generation after the addition of fondaparinux was comparable between plasma from patients with Child A and B cirrhosis (49.0 [44.1–55.3]% and 44.2 [19.4–58.7]% reduction in peak thrombin generation in the presence of TM, respectively) and in plasma from healthy controls (54.4 [47.7–62.3]% reduction in peak thrombin generation). A reduced anticoagulant response to fondaparinux was seen in plasma from patients with Child C cirrhosis, with only 12.9 [8.6–22.5]% reduction in peak thrombin generation when tested in presence of TM (P<0.001). In addition, when tested in the presence of TM, the prolongation of the lag time was more extensive in plasma from Child C patients (25.6 [25.0–38.1]%) as compared to controls (19.8 [12.3–25.6]%; P<0.01).

**Figure 3:**
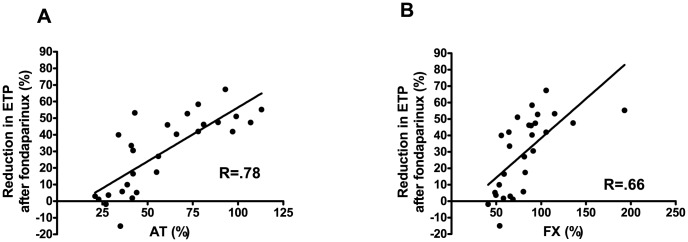
Correlation between AT/FX levels and reductions in thrombin generation after fondaparinux. Correlation between the percentual reduction in ETP when tested in the presence of TM after addition of 0.5 µg/ml fondaparinux and **(A)** plasmatic AT levels and **(B)** plasmatic FX levels in plasma from patients with cirrhosis.

Interestingly, reductions in peak thrombin generation by fondaparinux when tested in the presence of TM strongly correlated with both plasma antithrombin (AT) levels (r = 0.76, P<0.0001) and plasma factor X (FX) levels (r = 0.65, P<0.0001) (Figure 3).

### Rivaroxaban

Addition of rivaroxaban in the absence of TM hardly affected the ETP in both patients and controls. In contrast, peak thrombin generation was markedly reduced by rivaroxaban in both patients and controls, whereas lag times were prolonged. A reduced response to rivaroxaban was observed in plasma from cirrhotic patients as compared to control plasma, 46.5 [27.1–60.5]% peak reduction in patients vs. 61.8 [55.7–64.3]% peak reduction in controls in the presence of TM (P = 0.0005). In plasma from Child class C patients the lowest reduction in peak thrombin generation was observed, with a median reduction of 28.3 [21.1–39.5]%.

**Figure 4:**
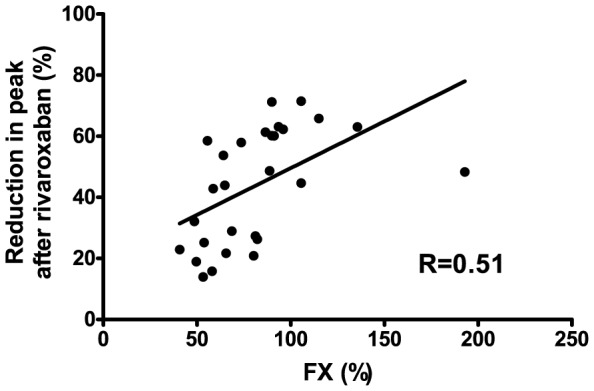
Correlation between FX levels and reductions in thrombin generation after rivaroxaban. Correlation between plasmatic FX levels and the percentual reduction in peak thrombin generation when tested in the presence of TM after addition of rivaroxaban at 25 ng/ml in plasma from patients with cirrhosis.

Interestingly, reductions in peak thrombin generation by rivaroxaban in the presence of TM correlated with plasma factor X (FX) levels (r = 0.61, P = 0.006) (Figure 4).

## Discussion

We observed a profoundly increased anticoagulant response to the direct thrombin inhibitor dabigatran in plasma from patients with cirrhosis compared to controls. The enhanced effect of dabigatran on thrombin generation was proportional to the severity of liver disease. Furthermore, we observed a modestly increased anticoagulant response to heparin and low molecular weight heparin in patients compared to controls, but only when tested in the absence of TM. In addition, a reduced response to fondaparinux and rivaroxaban was observed in plasma from patients with cirrhosis, and the reduced effect was also proportional to the severity of disease.

These data show that the anticoagulant potency of the different drugs differs substantially between cirrhotic patients and controls. In addition, the difference in anticoagulant potency between patients and controls differs substantially between the different drugs that were tested. The different drugs that were tested may also have an altered pharmacokinetics in patients with chronic liver disease, in particular in patients with advanced disease and in patients with concomitant renal failure. Therefore, dose adjustments based on both pharmacokinetics and altered anticoagulant potency of the specific drug may be required. Monitoring assays taking both drug level and drug potency would thus ideally be required for patients with liver diseases.

Although there is increasing clinical experience with heparins in patients with cirrhosis, clinical experience with NOACs in these patients is lacking. As we foresee that there might be an interest in using NOACs in patients with liver disease that require long-time anticoagulant treatment [19], we believe it is vital to understand the potentially altered behavior of these drugs in patients with liver disease, in addition to altered pharmacokinetics. We have recently demonstrated that, in contrast to heparins, monitoring of plasma levels of NOACs is possible in patients with liver disease [18], which will enable careful monitoring of these drugs. How altered drug potency needs to be assessed in clinical practice requires further study, but one could imagine that dose-adjustments based on plasma levels of FII (for Dabigatran) or FX (for Rivaroxaban) may be applicable given the linear relation between factor levels and anticoagulant potency (figures 2 and 4).

Our results extend recent observations by Senzolo et al. [17] on the increased anticoagulant response to LMWH in patients with cirrhosis. Our results, however, showed a more modest increase in anticoagulant potency of LMWH in patients with cirrhosis as compared to the data by Senzolo et al, and we failed to detect a difference in anticoagulant potency when thrombin generation was tested in the presence of TM. Indeed, clinical data on the use of heparins indicate that the drug is safe and effective in both the treatment and prevention of portal vein thrombosis [13], [14], [22]. The partially divergent results between our study and that of Senzolo et al. may be explained by the differences in LMWH dosage used, differences in patient characteristics, and slight differences in methodology. In fact, research has shown that thrombin generation is influenced substantially by preanalytical conditions [23], among which the protocol for centrifugation of blood to obtain platelet poor plasma. Senzolo et al. prepared the platelet poor plasma by double centrifugation at 2000 g for 10 min. In our study the second centrifugation step was set at 10.000 g as recommended [23]. Another possible critical difference in the method was the type and concentration of thrombomodulin used in the thrombin generation experiments.

Various anticoagulant drugs show different effects on the thrombin generation curves [24]. For example, direct factor Xa inhibitors substantially reduce the peak thrombin generation, while hardly affecting the endogenous thrombin potential, which was also observed with the direct factor Xa inhibitor rivaroxaban in this study. Therefore it has yet to be studied which parameter (ETP, peak, lag time, or velocity index) forms the best representation of the anticoagulant effect of a specific drug. In addition, the effects of a drug on the various parameters of the thrombin generation curve are not per definition concordant. For example, the reductions in peak and ETP after addition of fondaparinux and rivaroxaban were less pronounced in plasma from Child C cirrhotic patients compared to controls, which may imply increase in the dosages. However, Child class C patients exhibited the greatest prolongation of the lag time after the addition of both fondaparinux and rivaroxaban, which may imply that in patients with cirrhosis the dosage should be decreased.

We chose the concentrations of the various drugs to obtain appreciable (but not maximal) inhibition of thrombin generation in controls. The drug levels therefore do not necessarily represent peak levels found in clinical practice, although the levels we chose are all compatible with drugs levels at some stage of therapy. We would like to stress that the aim of our study was to provide proof of concept of differential drug potency in patients with liver disease rather than an exact estimate of drug potencies at clinically relevant plasma levels.

In conclusion, using *in vitro* thrombin generation assays, we observed a substantially increased anticoagulant response to dabigatran and a modestly increased anticoagulant response to heparin and LMWH in plasma from patients with cirrhosis. In addition, a reduced response to fondaparinux and rivaroxaban was observed in plasma from patients with cirrhosis. These results may imply that drug-specific dose adjustments may be required for patients with cirrhosis, in particular with dabigatran. However since the pharmacokinetics of the drugs may also be altered in these patients, the final dosing regimen should ideally take both the pharmacokinetics of the drug and the altered anticoagulant potency into account. Clinical studies on the *in vivo* effect of the available anticoagulant drugs in cirrhotic patients with thrombosis are needed to further validate this hypothesis.
